# Bilirubin neurotoxicity is associated with proteasome inhibition

**DOI:** 10.1038/cddis.2017.274

**Published:** 2017-06-15

**Authors:** Hongbiao Huang, Mingxing Guo, Ningning Liu, Chong Zhao, Haoyu Chen, Xiaoli Wang, Siyan Liao, Ping Zhou, Yuning Liao, Xin Chen, Xiaoying Lan, Jinghong Chen, Dacai Xu, Xiaofen Li, Xianping Shi, Li Yu, Yuqiang Nie, Xuejun Wang, Chang-E Zhang, Jinbao Liu

**Affiliations:** 1 Protein Modification and Degradation Lab, SKLRD, School of Basic Medical Sciences, Affiliated Cancer Hospital of Guangzhou Medical University, Guangdong, People’s Republic of China; 2 Guangzhou Institute of Cardiovascular Disease, The Second Affiliated Hospital, Guangzhou Medical University, Guangdong, People’s Republic of China; 3 Department of Pediatrics, Guangzhou First People's Hospital, Guangzhou Medical University, Guangdong, People’s Republic of China; 4 Department of Gastroenterology and Hepatology, Guangzhou Digestive Diseases Center, Guangzhou First People's Hospital, Guangzhou Medical University, Guangdong, People’s Republic of China; 5 Division of Basic Biomedical Sciences, Sanford School of Medicine of the University of South Dakota, Vermillion, SD, USA

## Abstract

The molecular mechanism underlying bilirubin neurotoxicity remains obscure. Ubiquitin–proteasome system-mediated proteolysis is pivotal to virtually all cellular processes and cell survival. Here we report for the first time that bilirubin at a clinically relevant elevated level impairs proteasomal function via inhibiting both the 19S proteasome-associated deubiquitinases (USP14 and UCHL5) and the chymotrypsin-like (CT-like) peptidase activity of 20S proteasomes, thereby contributing to bilirubin neurotoxicity. This is supported by multiple lines of evidence. First, sera from patients with hyperbilirubinemia were able to inhibit the peptidase activity of purified 20S proteasome *in vitro* in a bilirubin concentration-dependent manner; meanwhile, the blood cells of these patients showed significantly increased levels of ubiquitinated proteins (Ub-prs), consistent with proteasome inhibition. Second, intracerebroventricular injection to adult rats or intraperitoneal injections to neonatal rats of bilirubin-induced neural accumulation of Ub-prs, concurrent with other neural pathology; and brain malfunction and pathology induced by neonatal exposure to hyperbilirubinemia were detectable in the rats during their adulthood. Third, in primary cultures of hippocampal neurons, bilirubin strikingly induced Ub-pr accumulation before the activation of cell death pathway becomes discernible. Finally, bilirubin *in vitro* directly inhibited both the deubiquitination activity of proteasome-associated USP14 and UCHL5 and the CT-like peptidase activity of purified 20S proteasomes, in a dose-dependent manner. Hence, this study has discovered that increased bilirubin at a clinically achievable level can act as a proteasome inhibitor via targeting the 19S proteasome-associated deubiquitinases (DUBs) and, perhaps to a less extent, the 20S proteasome, identifying a novel mechanism for bilirubin neurotoxicity.

The pioneering studies of Stocker *et al.*^1, 2^ introduced the concept that unconjugated bilirubin (UCB) at low, ‘physiological’ plasma concentrations has a beneficial role by acting as a potent antioxidant that scavenges peroxyl radicals as efficiently as *α*-tocopherol. Despite this antioxidant behavior, if produced in excess, such as during hemolytic anemia or sepsis, UCB becomes neurotoxic through multiple mechanisms. Bilirubin encephalopathy is a subject of great clinical importance, but its pathogenesis at the molecular level is not fully understood.^3^ Early studies established that mitochondria may be a major target for UCB neurotoxicity, as demonstrated by impairment in mitochondrial function leading to the uncoupling of oxidative phosphorylation.^4, 5, 6, 7, 8^ Additional effects of UCB in neural tissues and neuronal cell lines include inhibition of DNA and protein synthesis, changes in carbohydrate metabolism, and modulation of neurotransmitter synthesis and release. Most of the early neural toxicity data were obtained in cell cultures using excessively high UCB concentrations, exceeding its very low aqueous solubility^9^ and the high-affinity binding capacity of plasma albumin.^10^ Moreover, the source of the albumin or plasma that is often used in the binding experiments is adult blood; however, plasma from newborns has a diminished binding capacity for UCB.^11, 12^ These factors potentially diminish the clinical relevance of some of the previously published *in vitro* toxicity findings to the *in vivo* conditions prevalent in the majority of neonatal jaundice.^13, 14^

The ubiquitin–proteasome system (UPS) degrades individual proteins in the cell. The 26S proteasome consists of a barrel-shaped 20S core particle capped by the 19S regulatory complex. The proteasomal peptidase activities reside in the 20S proteasome while several deubiquitinating enzymes (DUBs, e.g., RPN11, USP14 and UCHL5), which function to shorten or removal ubiquitin chains, are known to associate with the 19S proteasome. Proper functioning of the UPS is essential to the function and the survival of the cell and proteasome malfunction can be lethal to the cell.^15^ To date, no reported study has examined the effect of bilirubin on UPS.

Here we report that bilirubin at a clinically relevant concentration is capable of inhibiting UPS-mediated protein degradation via acting as an endogenous inhibitor of proteasomal DUBs and of the 20S proteasome; we also provide the first evidence that the proteasome inhibition property of bilirubin very likely contributes to its neurotoxicity.

## Results

### Elevated levels of serum bilirubin inhibit proteasome function

**Figure 1:**
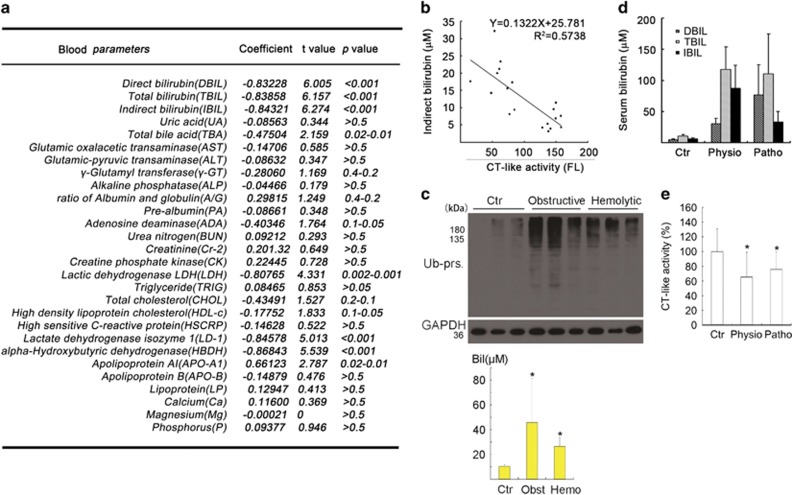
Inhibition of proteasome peptidase activity by human sera in a serum bilirubin level-dependent manner. (**a**) Correlation analyses between the level of individual chemical constituents in sera from 10 patients with digestive diseases and the *in vitro* effect of the sera on the CT-like peptidase activity of purified human 20S proteasomes. (**b**) Inverse correlation between the serum indirect bilirubin level and the CT-like activity of the serum treated 20S proteasomes. (**c**) Western blot analyses for Ub-prs in the blood cells from normal control (Ctr) humans and patients with obstructive (Obst) and hemolytic (Hemo) jaundice, with their serum bilirubin concentration summarized in the associated bar graph. Mean±S.D., *n*=3; *P<0.05 *versus* Ctr. (**d**) Serum bilirubin concentrations in human control infants and the infants with physiological (Physio) or pathological (Patho) jaundice. Mean±S.D. (*n*=10 per group). (**e**) Comparison of the effects of sera from controls and infants with physiological and pathological jaundice on proteasomal CT-like activity. Mean±S.D. (*n*=10 per group). *P<0.05 *versus* Ctr

In adult patients, increased serum bilirubin is more frequently seen in patients with hepatic or bile duct diseases than other types of patients. Hence, we collected blood samples from patients with digestive disease and, from these samples, serum and blood cells were obtained. To explore the effect of serum bilirubin on proteasome function, human 20S proteasomes were incubated with the serum of patients with digestive disease for 1 h in the presence of a specific fluorogenic substrate for proteasomal chymotrypsin-like (CT-like) activity. Linear correlation analysis between a panel of biochemical parameters and the proteasomal CT-like activity assessed with these serum samples revealed that the assessed proteasome peptidase activities were negatively correlated to the levels of serum bilirubin, including direct (DBIL), indirect (IBIL) and total bilirubin (TBIL), and to the serum levels of total bile acid (TBA) and of liver enzymes/proteins (LDH, LD-1, HBDH and APO-A1) that reflect liver injury (Figures 1a and b). The results imply that elevated serum bilirubin from patients with digestive disease may be able to inhibit the 20S proteasome. Therefore, we measured the level of total ubiquitinated proteins (Ub-prs) in the blood cells of patients with clinically diagnosed jaundice, using western blot analysis. We found that it was significantly increased in the blood cells of patients with jaundice, especially those with obstructive jaundice and with higher levels of bilirubin compared with normal people and hemolytic jaundice patients (Figure 1c). To further single out the effect of increased bilirubin levels, we also collected sera from normal control neonates and from the neonates with physiological or pathological jaundice to test their effects on the proteasome peptidase activity of purified 20S proteasomes *in vitro*. The results showed that sera from newborns with either physiological or pathological jaundice were also able to similarly inhibit proteasome peptidase activity (Figures 1d and e). Taken together, these results from the tests using human materials are fully consistent with a novel notion that bilirubin can suppress proteasomal protein degradation.

### Bilirubin treatment inhibits UPS function

**Figure 2:**
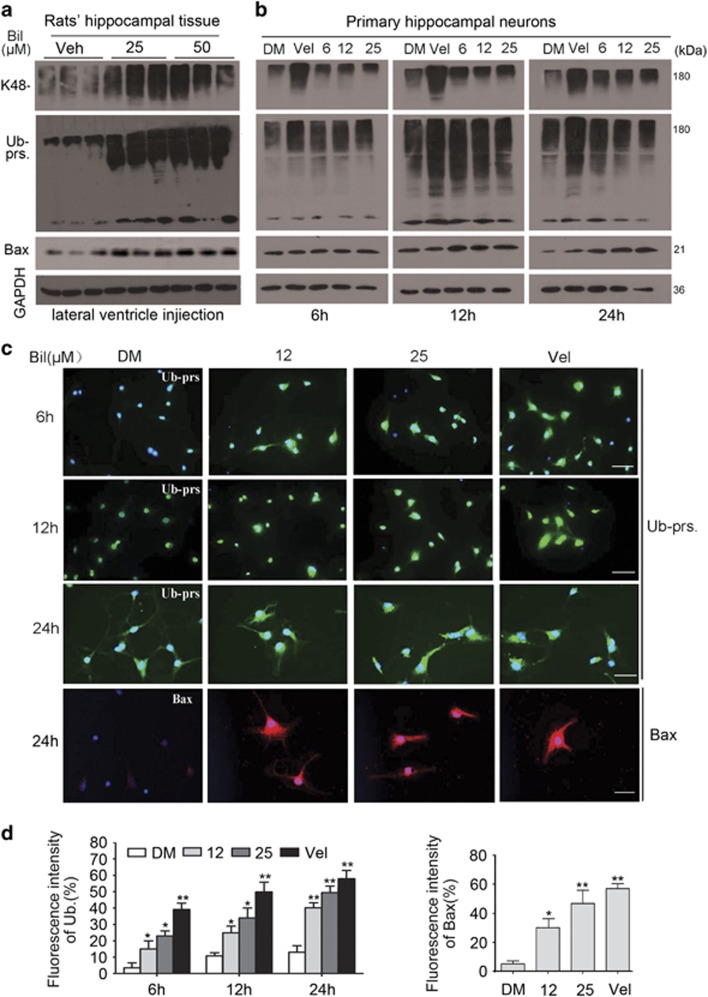
Bilirubin inhibits the UPS. (**a**) Immunoblotting analyses for K48-linked Ub-prs (K48-Ub-prs.), total Ub-prs and Bax in the hippocampal tissue of adult rats that were subject to intracerebroventricular injection of 10 *μ*l of bilirubin (Bil, 25 or 50 *μ*mol/l) or vehicle control (Veh). Tissue sample was collected at 24 h after bilirubin injection into the lateral ventricle. (**b**) Western blot analyses for K48-Ub-prs, Ub-prs and Bax in primary neurons cultured with the indicated doses of bilirubin for 6, 12 and 24 h. DM, DMSO; Vel, bortezomib (50 nM). (**c**) Photomicrographs of immunofluorescence staining for Ub and Bax in primary hippocampal neurons treated with the indicated concentration of bilirubin for the indicated times. DM, DMSO; Vel, bortezomib (50 nM). Scale bar=20 *μ*m. (**d**) Analysis of imunofluorescence intensity of ubiquitin (Ub) and Bax from the experiment illustrated by panel (**c**). *P<0.05 and **P<0.01 *versus* the control (DM)

**Figure 3:**
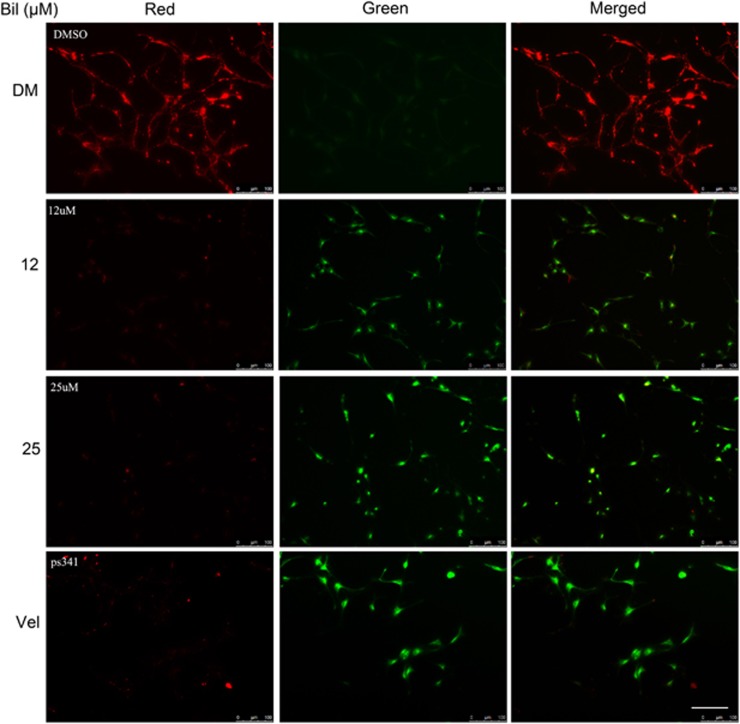
Bilirubin diminished mitochondrial membrane potentials in primary neuronal cells. Hippocampal primary neurons were treated with different concentrations (DMSO, 12, 25 *μ*M) of bilirubin for 24 h and mitochondrial membrane potential (ΔΨm) was detected by photomicrographs of JC-1 fluorescence staining. Scale bar=100 *μ*m. Red fluorescence represents the mitochondrial aggregate form of JC-1, indicating intact mitochondrial membrane potential. Green fluorescence represents the monomeric form of JC-1, indicating dissipation of ΔΨm

We next sought to determine the sufficiency of elevated bilirubin to induce UPS malfunction in animal brains and the primary cultures of neurons. The degradation of Ub-prs, especially those with a polyubiquitin chain formed with ubiquitin lysine 48 (K48) linkage, generally relies on the 26S proteasome; thus, accumulation of total or K48-linked Ub-prs in the cell is often used as an indicator of reduced proteasomal function. We treated adult rats with bilirubin via intracerebroventricular injection and then extracted the hippocampal tissue for western blot analyses. We found that proapoptotic protein Bax, as well as total and K48-linked Ub-prs were significantly increased in the hippocampus by the bilirubin treatment (Figure 2a). To examine the direct effect of bilirubin on hippocampal neurons, we also treated cultured primary hippocampal neurons with different doses of bilirubin (6, 12, 25 *μ*M) for a duration of 6, 12 or 24 h by using Velcade as a positive control for proteasome inhibition.^15, 16^ Western blot analyses of the cell lysates revealed that Ub-prs in the cultured primary hippocampal neurons were increased by bilirubin treatment at a dose as low as 6 *μ*M after 6 h of treatment; and the increase was most pronounced after 12 h of treatment (Figures 2b–d). We also observed an increase in proapoptotic protein Bax in bilirubin-treated cells but the increase of Bax did not become discernible until 12 h after bilirubin treatment was initiated (Figure 2b), indicating that proteasome impairment occurs earlier than activation of the apoptotic pathway in the bilirubin-treated hippocampal neurons. Activation of cell death pathways is often associated with dissipation of mitochondrial membrane potential (ΔΨm); hence, we tested the impact of bilirubin on ΔΨm. We found that ΔΨm was also diminished in a dose-dependent manner by bilirubin treatment (Figure 3). Taken together, these results suggest that proteasome inhibition is induced by bilirubin and may contribute to the induction of neuronal apoptosis by bilirubin.

Immunofluorescence microscopy was also conducted to examine the distribution of Ub-prs and proapoptotic protein Bax in cultured hippocampal neurons treated with bilirubin for 6, 12 and 24 h. It was revealed that accumulation of Ub-prs was promoted by bilirubin in a dose- and time-dependent manner. Significant Ub-prs accumulation was observed in cells treated with 12 and 25 *μ*M of bilirubin for 24 h. Bilirubin at 12 *μ*M and bortezomib (Velcade, Vel) at 50 nM showed a similar level of Ub-pr accumulation (Figures 2c and d). Taken together, these findings indicate that bilirubin can inhibit UPS proteolytic function in hippocampal neurons and induce neuronal apoptosis, and also suggest that the UPS inhibition likely contributes to the induction of neuronal toxicity by bilirubin.

### Bilirubin inhibits proteasomal DUBs

**Figure 4:**
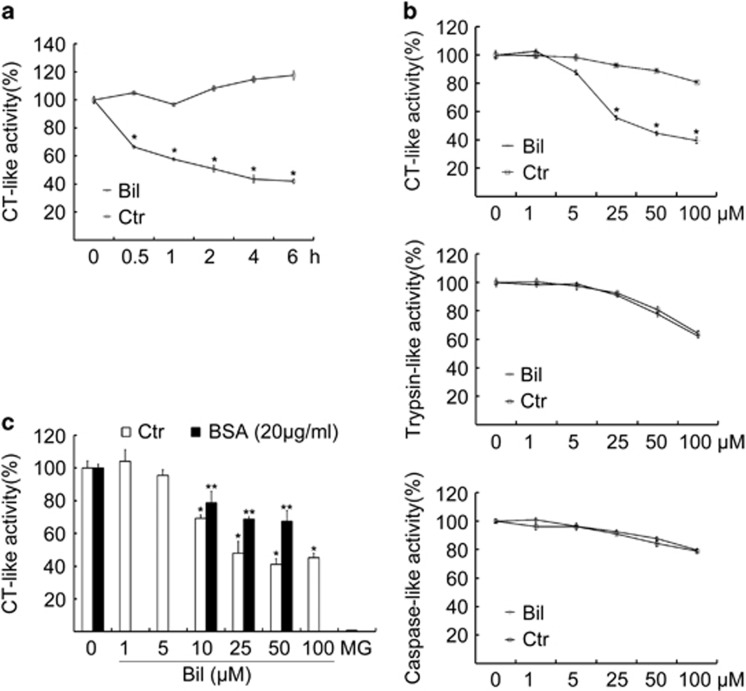
Bilirubin inhibits proteasome peptidases. (**a**) The effect of bilirubin on proteasome peptidase activity of primary neurons in cultures. Primary neuronal cells were treated with bilirubin (Bil, 25 *μ*M) or vehicle control (Ctr) for 6 h, followed by addition of proteasome substrates to the treated cells, and then the CT-like peptidase activities were detected. Mean±S.D. (*n*=3), *P<0.05 *versus* Ctr. (**b**) The effect of bilirubin on proteasome peptidase activities *in vitro*. Purified human 20S proteasomes were treated with the indicated doses of bilirubin or vehicle control *in vitro* and then proteasome peptidase activities were detected. Mean±S.D. (*n*=3), *P<0.05 *versus* Ctr. (**c**) The effect of bilirubin and BSA on proteasome CT-like peptidase activities *in vitro*. Purified human 20S proteasomes were treated with the indicated doses of bilirubin or vehicle control *in vitro* in absence or presence of BSA and then proteasome CT-like peptidase activities were detected. Mean±S.D. (*n*=3), *P<0.05 *versus* the Ctr without Bil treatment; **P<0.05, compared with Bil treatment alone

**Figure 5:**
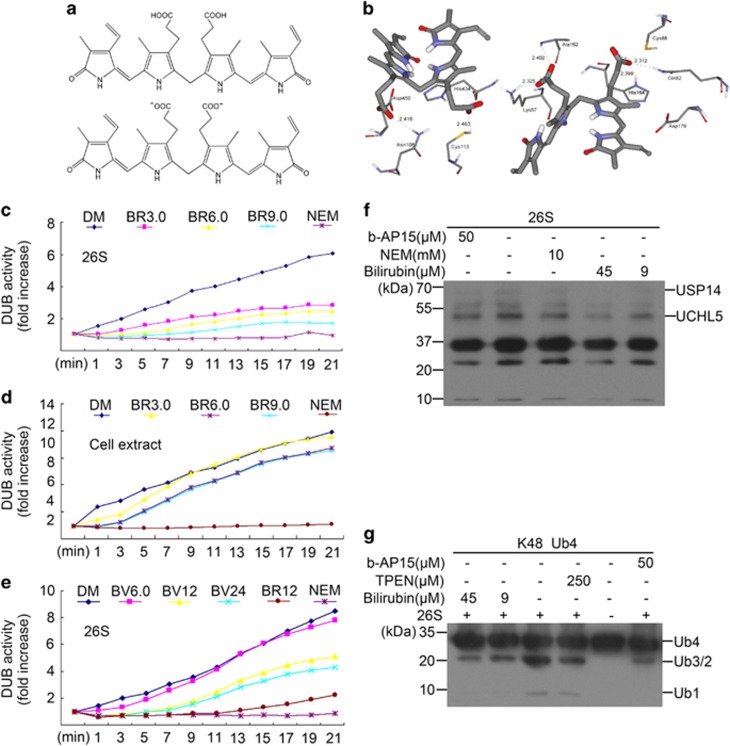
Bilirubin selectively inhibits proteasomal DUBs. (**a** and **b**) Computational molecular docking of bilirubin with USP14 and UCHL5. The binding modes of bilirubin anion (**a**) and its binding mode at the active site of USP14 and UCHL5 (**b**) are shown. For DUB activity assays shown next, a fluorogenic substrate Ub-AMC was used. (**c**) The effect of bilirubin on proteasomal DUB activity. Purified human 26S proteasomes were treated with vehicle control DMSO (DM) or bilirubin (BR) at 3, 6 or 9 *μ*M (BR3.0, BR6.0, BR9.0, respectively) or with NEM (2 mM); the DUB activity at different times was then recorded. (**d**) The effect of bilirubin on total DUB activity. Cell lysates from primary neurons were treated with the indicated doses of bilirubin or NEM (2 mM), then the DUB activity at different times was recorded. (**e**) The effect of biliverdin (BV) on proteasomal DUB activity. Purified human 26S proteasomes were treated with the indicated doses of BV (*μ*M) or with BR (12 *μ*M) or NEM (2 mM), then the DUB activity at different times was recorded. (**f**) Active site–directed labeling of proteasomal DUBs. Purified human 26S proteasomes were treated with bilirubin, b-AP15 or NEM for 10 min before labeled with HA-UbVS and fractionated via SDS-PAGE. The covalently bound HA-UbVS was detected by western blot for the HA tag. (**g**) Ub chain disassembly assay. K48-linked Ub tetramers (K48-Ub4) were disassembled by the purified human 26S proteasomes after treatment with bilirubin, b-AP15 or TPEN

To decipher the target of bilirubin in the UPS, we examined the effect of bilirubin treatment on 20S proteasome peptidase activities *in vitro*. We found that bilirubin treatment at a dose of 25 *μ*M inhibited proteasome CT-like peptidases in live neuron cells within 30 min (Figure 4a). However, *in vitro* assays showed that bilirubin at a concentration up to 10 *μ*M could not inhibit the activities of the purified 20S proteasome CT-like peptidases; the IC50 value for bilirubin to inhibit the CT-like activity was ~20 *μ*M (Figure 4b), which is, notably, below the average serum bilirubin levels observed in newborn humans with physiological and pathological jaundice (Figure 1d) and adult patients with either obstructive or hemolytic jaundice (Figure 1c). Bilirubin at a concentration up to 100 *μ*M did not show significant effect on the trypsin-like and caspase-like activities of the proteasome (Figure 4b). In addition, we used bovine serum albumin (BSA), which binds to and sequesters free bilirubin, to test its effect on bilirubin-induced inhibition of proteasome CT-like activities. We found that BSA treatment (20 *μ*g/ml) by itself showed no effect on the CT-like activity but was able to rescue the proteasome inhibition induced by a finite amount of bilirubin (25, 50 *μ*M) (Figure 4c). Comparing the *in vivo* (Figure 2b) and *in vitro* (Figure 4b) effects of bilirubin on the proteasome, we noted an apparent discrepancy that the minimal effective dose for bilirubin to induce discernible accumulation of Ub-prs in cultured primary hippocampal neurons (Figure 2b) is 6 *μ*M, a dose that was not very effective in inhibition of purified 20S proteasomes *in vitro* (Figures 4b and c). This discrepancy prompted us to hypothesize that in addition to directly inhibiting 20S proteasome CT-like activity, bilirubin may also inhibit other activities of the 26S proteasome, such as the DUB activity of the 26S proteasome. The proteasome DUB activity is attributed to the action of USP14, UCHL5 and RPN11 located in the 26S proteasome. USP14 and UCHL5 are both cysteine DUBs. Previous studies have revealed that the catalytic core of USP14 is formed by Cys113, His434 and Asp450, and that of UCHL5 is formed by Cys88, His164 and Asp179.^17, 18^ Our molecular docking studies unveiled that bilirubin anion could strongly bind to each active site of the two DUBs (USP14 and UCHL5), with a CDOCKER Interaction Energy of −35.16 and −38.04 kcal/mol, respectively. Accordingly, the binding modes of bilirubin anion are displayed in Figure 5, in which bilirubin anion is suitably situated at the entrance of each active site. In addition, bilirubin anion has a size large enough to prevent other substrate from entering into the active pocket. Moreover, bilirubin anion can form several hydrogen bonds with the two DUBs, which may further enhance the binding affinity. In the binding pocket of USP14, two O atoms of bilirubin anion can potentially form two hydrogen bonds with Asn108 and Cys113, with corresponding bond lengths of 2.418 and 2.463Å. In the binding site of UCHL5, four hydrogen bonds between three O atoms of bilirubin anion and four side chains, Gln82, His164, Lys57 and Ala162 could form, with their bond lengths being respectively 2.312, 2.399, 2.325 and 2.402Å. As bilirubin anion can bind to the catalytic cores of the two DUBs through steric effect and hydrogen bonds, the catalysis of the DUBs will be inhibited in the presence of bilirubin anion (Figures 5a and b). The following experiments were performed to verify this computational model.

Next, we assessed the effect of bilirubin on 26S proteasomal DUBs using a fluorogenic substrate Ub-AMC (ubiquitin conjugated aminomethylcoumarin). Similar to DUB inhibitor *N*-ethylmaleimide (NEM), bilirubin (3, 6 and 9 *μ*M) showed substantial inhibition on the DUB activities of purified 26S proteasomes (Figure 5c). As shown in Figure 5d, unlike NEM, which is a general inhibitor of cysteine proteases and completely blocked the total DUB activities in the cell lysate, bilirubin (3, 6 and 9 *μ*M) yielded weak or virtually no effect on the DUB activities in cell lysates. This result indicates that bilirubin is a specific inhibitor of the proteasomal DUBs. Biliverdin, which is the precursor of bilirubin and shares some chemical structure similarities to bilirubin, also showed an inhibitory effect on the 26S proteasomal DUB activities but the effect was much weaker, compared with bilirubin at the same concentration (Figure 5e). These results indicate that the conversion from biliverdin to bilirubin by biliverdin reductase significantly enhances the 26S proteasome DUBs inhibitory property of these heme metabolites. We further performed proteasomal DUB active site labelling tests using HA-Ubiquitin-Vinyl Sulfone (HA-UbVS). HA-UbVS is a potent inhibitor against, and can bind covalently to, the active site of UCHL5 and USP14 associated with the 26S proteasome. We found that bilirubin (9 and 45 *μ*M) was able to compete with HA-UbVS for binding both UCHL5 and USP14 (Figure 5f). The cleavage of tetraubiquitin chains (Ub4) mediated by the 26S proteasome DUBs was also tested to further confirm this effect. As shown in Figure 5g, K48-linked tetraubiquitin chains (K48-Ub4) were cleaved in the presence of 26S proteasomes and this was efficiently blocked by bilirubin (9 and 45 *μ*M), more effectively than by b-AP15 (a known UCHL5/USP14 inhibitor) and TPEN (a zinc chelator known to inhibit the JAMM family of DUBs, to which RPN11 of the 26S proteasome belong). Taken together, these results show that bilirubin targets 19S proteasome-associated DUBs.

### Bilirubin inhibits neurite growth *in vitro* and induces hippocampus neuron loss *in vivo*

**Figure 6:**
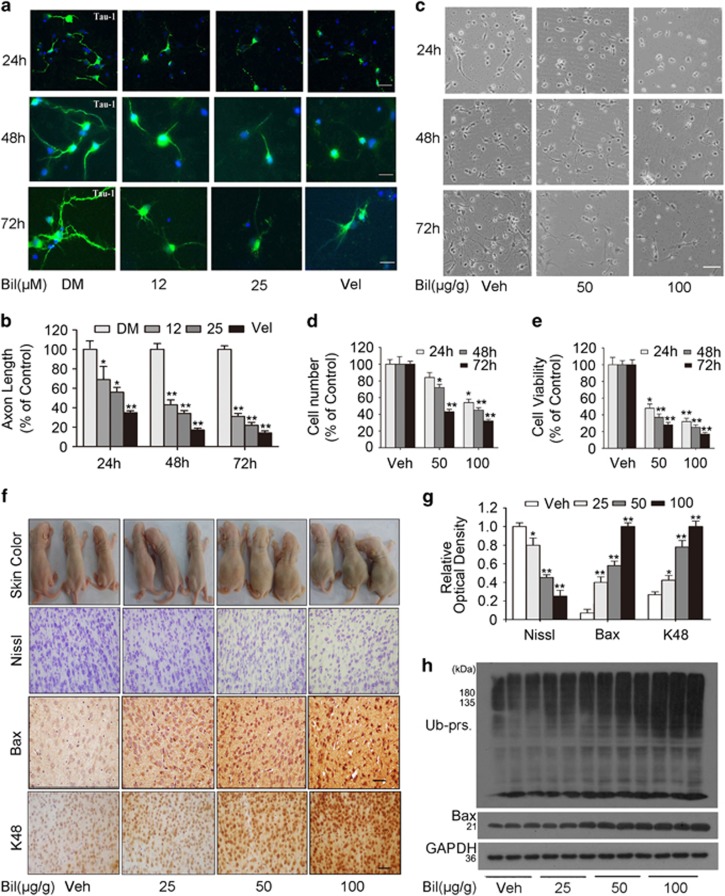
Bilirubin inhibits neurite growth and induces hippocampal neurons loss. (**a**) To observe inhibition effect of bilirubin on the growth of axons and measure the length of axons, primary hippocampal neurons were treated with either DMSO (DM) or different concentrations (12, 25 *μ*M) of bilirubin for 24, 48 and 72 h, then immunofluorescence staining were carried out with the Tau-1 antibody (marked neuron body and axon). Treatment with a *bona fide* proteasome inhibitor Velcade (Vel) was included as a positive control. Bar=20 *μ*m. (**b**) Statistical graph of axon length of primary hippocampal neurons at corresponding time point. (**c**) 24 h after two intraperitoneal injections into newborn SD rats with bilirubin (50 or 100 *μ*g/g), hippocampal were extracted from newborn rats to conduct primary neuron cell culture. Phase contrast images of the cultured neurons were taken at 24, 48, and 72 h after plating; Bar=20 *μ*m. (**d** and **e**) Statistical graph of the cell number and the cell viability at different time point. (**f**–**h**) Newborn rats were photographed to show their skin color at 3 days after being subject to intraperitoneal injections of the indicated doses of bilirubin (two times per day for 3 days); the animals were then killed and brain tissues were sampled for histochemical detection of the Nissl bodies, immunohistochemistry staining for Bax and K48-linked Ub chains (**f** and **g**), or western blot analyses for total Ub-prs and Bax (**h**). Bar=100 *μ*m, (**g**) quantification of Nissl, Bax and K48-linked Ub chains in brain tissues. *P<0.05, **P<0.01 *versus* the control group

The primary hippocampal neuron is a well-defined model for studying axon development.^19^ To study the toxic effect of bilirubin on neurons, we exposed primary hippocampal neurons in cultures to different concentrations of bilirubin for 1, 2 and 3 days, followed by immunofluorescence staining for tau-1, a marker of neuron and axon.^20^ By using Image-Pro Plus software (Media Cybernetics, Inc., Rockville, MD, USA) to measure and analyze quantitatively the length of axons,^21^ we found that bilirubin displayed a dose-dependent suppressive effect on neuronal growth and axon formation (Figures 6a and b). In addition, we treated newborn rats with intraperitoneal injections of bilirubin (50 and 100 *μ*g/g, twice a day for 1 day), and conducted primary culture of cells extracted from the hippocampal tissue of these rats, lasting for 1–3 days. By counting the cell number and measuring the cell viability with MTS, we found that the growth of hippocampal neurons from the bilirubin-treated rats was significantly suppressed (Figures 6c–e).

Moreover, we administered bilirubin to newborn rats via intraperitoneal injections (25, 50 or 100 *μ*g/g, twice a day for 3 days) to directly examine *in vivo* effects. We found that rats’ jaundice became more pronounced as the injected bilirubin concentrations increased. HPLC detection of blood bilirubin concentrations showed that 25, 50 and 100 *μ*g/g of bilirubin injected for six times induced hyperbilirubinemia in rats (data not shown). Histochemical examination showed decreases of Nissl bodies and increases of proapoptotic protein Bax and K48-linked Ub-prs in the brain by bilirubin treatment in a dose-dependent manner (Figures 6f and g). The increases of Bax and the accumulation of Ub-prs in bilirubin-treated brain were further verified by western blot analyses (Figure 6h). These *in vivo* findings further demonstrate that an increase in circulating bilirubin is sufficient to suppress neural proteasome function and induce neuronal loss.

### Neonatal exposure to increased bilirubin induces cognitive impairment and neuron loss in later life

**Figure 7:**
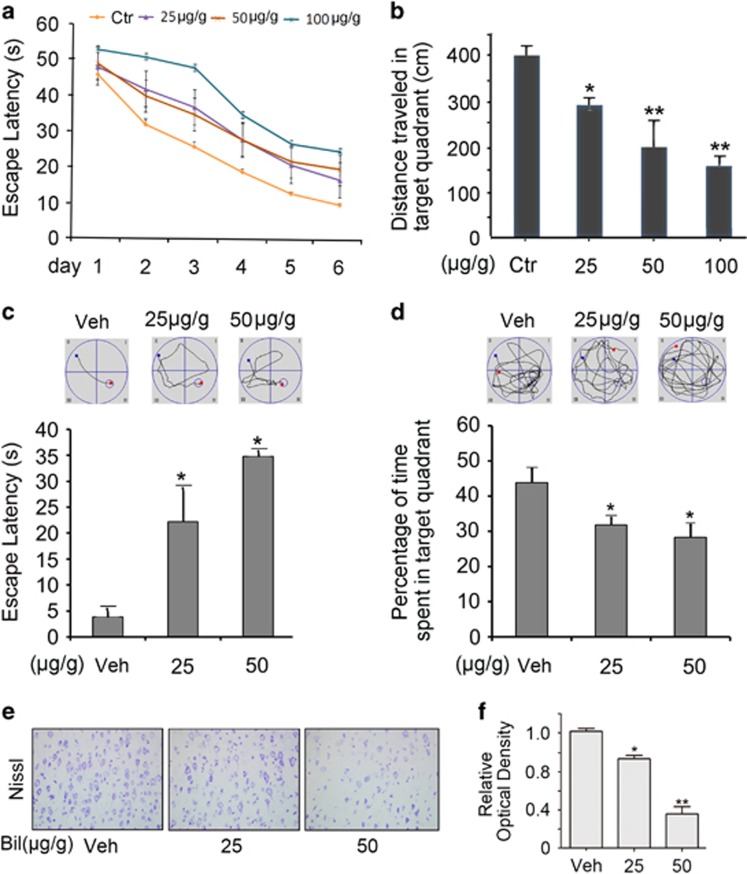
Bilirubin treatment impairs the spatial learning and memory. Morris water maze tests were performed on rats at 1 month (**a** and **b**) and 4 months (**c** and **d**) after they were intraperitoneally injected with saline (vehicle, Veh) or the indicated doses of bilirubin twice per day for 3 days. (**a**) The effect of bilirubin treatment on the escape latency of the rats at 1 month after treatment. (**b**) The effects of bilirubin treatment on the distance traveled in the target quadrant in 60 s after the platform was withdrawn. (**c** and **d**) Representative trajectory charts and the summary of the escape latency data of the rats at 4 months after treatment, (**c**) and of the duration of time traveled in the target quadrant (**d**) are shown to illustrate changes in the ability of spatial memory. *P<0.05, **P<0.01 *versus* the control group (Ctr); *n*⩾8 rats for each group. (**e** and **f**) Newborn rats were treated with bilirubin as **c** and **d**, the animals were then killed at 4 months of age and brain tissues were sampled for histochemical detection of the Nissl bodies. Representative images (**e**) and quantitative data (**f**) are shown. Bar=100 *μ*m. *P<0.05, **P<0.01 *versus* the vehicle (Veh) control group

The water maze test was performed on 1 month and 4 months old rats who had been subject to intraperitoneal injections of different doses of bilirubin (N.S, 25, 50 and 100 *μ*g/g) twice per day for 3 days when they were newborns. By 1 month after the bilirubin injections, all rats were trained for 6 consecutive days to remember the location of the platform. The data showed that the latency (the time to find location of the platform) was significantly increased for bilirubin-treated rats relative to the control rats, which indicates a bilirubin-induced decrease of learning capability (Figure 7a). When platform was removed, rats’ memory was tested. The results showed that bilirubin-injected rats stayed less time and traveled less distance in the target quadrant than the control group (*P*<0.05 or 0.01, Figure 7b), which unraveled the fact that bilirubin could induce a decrease in rats’ memory. When grown up to 4 months of age, the bilirubin-treated rats (25, 50 *μ*g/g) displayed a significantly increased escape latency (Figure 7c) and significantly reduced percentage of time traveled in the target quadrant (Figure 7d), compared with the control rats. Notably, for the experiments at the 4 months of age, we did not include the 100 *μ*g/g bilirubin treatment group because an extreme high mortality of these mice occurred between 1 and 4 months after neonatal exposure to this dose of bilirubin. Overall, the data from the water maze tests demonstrate that neonatal exposure to elevated bilirubin can lead to impairment in the spatial learning and memory abilities of the animals in their adult life.

To test whether bilirubin exposure at the neonatal stage leads to brain pathological changes in adulthood, we performed histological staining for Nissl bodies in the brain of 4-month-old rats who, at their neonatal stage, had received the intraperitoneal injections of different doses of bilirubin (vehicle, 25 and 50 *μ*g/g) twice per day for 3 days. The histochemical results showed very striking decreases of Nissl bodies in the hippocampus of the bilirubin-treated rats (Figures 7e and f).

Taken together, these results show that hyperbilirubinemia can acutely inhibit UPS activities and its function, activate apoptosis and induce neurons loss, in both neonatal and adult rat brains and, strikingly, the pathological changes induced at the neonatal stage are long-lasting and clearly detectable after the rats fully grow up into their adulthood.

## Discussion

Bilirubin encephalopathy occurs in newborns with hyperbilirubinemia. UCB is a known neurotoxin; at abnormally high concentrations, it can cause permanent neurological damage in neonates.^22, 23, 24, 25^ Here we demonstrate for the first time that bilirubin can effectively inhibit proteasomal function through both direct inhibition of 19S proteasome-associated DUBs (USP14 and UCHL5) and suppressing 20S proteasome peptidase activity and that elevated levels of serum bilirubin are capable of suppressing proteasomal protein degradation in neurons *in vitro* and *in vivo*, providing a novel pathogenic mechanism for neonatal bilirubin encephalopathy.

In this study, we collected abundant evidence that elevated levels of bilirubin can lead to proteasome inhibition in neurons *in vivo* and in cell cultures. First, serum bilirubin levels of patients with digestive disease or neonatal physiological and pathological jaundice are positively correlated to the ability of the sera to inhibit the 20S proteasome peptidase activity *in vitro* (Figure 1); second, Ub-prs in the blood cells of patients with jaundice were significantly accumulated (Figure 1c); third, bilirubin treatment at a dose comparable or lower than the serum bilirubin levels of patients with jaundice was sufficient to significantly inhibit the proteasome in rat brains (Figure 2a) and cultured primary rat hippocampal neurons (Figures 2b–d); and finally, bilirubin could directly inhibit the CT-like activity of purified 20S proteasomes *in vitro* and this inhibition was diminished in the presence of excessive amount of BSA, which binds and sequesters bilirubin and thereby reduces the availability of free bilirubin to the proteasome (Figure 4c).

Moreover, we have demonstrated that proteasomal DUBs are key targets of bilirubin for its proteasome inhibition. This conclusion is compellingly supported by multiple lines of evidence presented here: (i) our molecular docking analyses predicted that the catalysis of the proteasomal DUBs (USP14 and UCHL5) could be inactive in the presence of bilirubin because bilirubin anion is predicted to strongly bind to the catalytic cores of the two DUBs through both steric effect and forming hydrogen bonds (Figures 5a and b); (ii) our DUB active site-directed labeling assays confirmed that bilirubin binds to the active sites of both USP14 and UCHL5 associated with 26S proteasomes (Figure 5f); (iii) the *in vitro* DUB activity of purified 26S proteasomes (Figure 5c), but not that of whole-cell lysates (Figure 5d), was effectively inhibited by incubation with bilirubin at a dose as low as 3.0 *μ*M; and (iv) our polyubiquitin chain disassembly assays showed that bilirubin effectively prevented the 26S proteasome from disassembling K48-linked tetraubiquitin chains *in vitro* (Figure 5g). Therefore, although our data collectively suggest that bilirubin exert proteasome inhibition potentially through both directly targeting the 19S proteasome-associated DUBs and directly or indirectly suppressing the CT-like peptidase of the 20S proteasome, the former is more likely the primary mechanism underlying the neurotoxicity of elevated bilirubin because the minimal effective concentration of bilirubin to accumulate Ub-prs in primary hippocampal neurons was much lower than its minimal effective concentration to inhibit purified 20S proteasome peptidase activity *in vitro*.

Along with others, we previously have been reported that inhibition of USP14 and UCHL5 of the 19S proteasome induces apoptosis.^26, 27, 28, 29^ In bilirubin-treated cells, Ub-prs were significantly accumulated at 6 h after bilirubin treatment, but the proapoptotic protein bax was not obviously increased until 12 h after bilirubin treatment, indicating that proteasome inhibition precedes apoptosis (Figures 2b–d). Mitochondrial membrane potential detected by JC-1 staining was diminished in a dose-dependent manner by bilirubin treatment (Figure 3). These changes are consistent with the ability of bilirubin to induce apoptosis observed in previous reports.^30, 31^ Furthermore, we observed in newborn rats that hyperbilirubinemia induced with intraperitoneal injections of bilirubin resulted in an increase in Ub-prs, concurrently with decreases of Nissl bodies and increases of Bax in the brain (Figure 6). We also found that rats showed decreased learning capacity and impaired memory long after (1 and 4 months tested) they had received bilirubin treatment at the neonatal stage (Figure 7). Strikingly, the decreases of Nissl bodies resulted from neonatal hyperbilirubinemia remain evidenced in the brain at 4 months after the bilirubin treatment (Figures 7e and f). Consistent with our report, several previous studies have showed that bilirubin-induced neurologic dysfunction including learning difficulties and loss of cognition in humans.^32, 33, 34^

In summary, here we have provided novel evidence that the newly discovered proteasome inhibition property of bilirubin potentially has an important role in bilirubin encephalopathy of neonates. Establishment of this new pathogenic pathway will have a broad and long-lasting impact on the mechanistic exploration and the treatment of a host of life-threatening and highly disabling disorders related to abnormal bilirubin metabolism.

## Materials and methods

### Materials

Bilirubin and biliverdin were purchased from Sigma-Aldrich Inc. (St. Louis, MO, USA). Other agents used include NEM (Sigma-Aldrich Inc.), the Proteasome-Glo Chymotrypsin-like Cell-Based Assay kit (Promega Bioscience, Madison, WI, USA), Suc-Leu-Leu-Val-Tyr-aminomethylcoumarin (Suc-LLVY-AMC), Boc-Leu-Arg-Arg- aminomethylcoumarin (Boc-LRR-AMC), Z-Leu-Leu-Glu-AMC, 20S and 26S human proteasome preparations, HA-Ub-VS, K48-linked tetraubiquitin, Ubiquitin-AMC (U550) (Boston Biochem, Cambridge, MA, USA), JC-1 (Beyotime, Shanghai, China) and MTS assay kit (CellTiter 96 Aqueous One Solution reagent) (Promega Corporation, Madison, WI, USA). Antibodies used in this study and their sources are: anti-ubiquitin (P4D1) (Santa Cruz Biotechnology Inc., Santa Cruz, CA, USA), anti-K48-linkage specific polyubiquitin (D9D5), anti-Bax (D3R2M) (Cell Signaling Technology, Beverly, MA, USA), anti-GAPDH, anti-HA-tag (Bioworld Technology, Inc., Louis Park, MN, USA) and anti-Tau-1, (Invitrogen, Carlsbad, CA, USA). Enhanced chemiluminescence (ECL) reagents were purchased from Santa Cruz Biotechnology Inc.

### Bilirubin administration

The protocol for the care and use of all animals in this study was in accordance with the Guangdong Animal Center for the ethical treatment of animals and approved by the Institutional Animal Care and Use Committee of Guangzhou Medical University (Guangzhou, China). Sprague-Dawley rats (Grade SPF) were obtained from Guangdong Laboratory Animal Monitoring Institute. All rats were kept in cages under a 12-h light : 12-h dark cycle with the light on from 0700 to 1900 hours. Animal housing and bilirubin treatment were done at a stable temperature (23–25 °C) and humidity. All animals had access to standard laboratory diet and drinking water *ad libitum*, and newborn rats were breast fed.

A cohort of newborn rats of both sexes at postnatal day 3 were randomly divided to six experimental groups by litter and body weight, and were intraperitoneally injected with physiological saline (control group) or 6, 12, 25, 50 and 100 *μ*g/g bilirubin, respectively, twice every day for 3 days. All rats were killed 12 h after the final injection, for analyses. The 25 or 50 *μ*g/g bilirubin injection regime caused significant hyperbilirubinemia; hence, we administered the dosage of 25 and 50 *μ*g/g bilirubin injection regimes to another cohort of newborns rats for follow-up studies to test the change of brain function at 1 and 4 months of age.

Adult male rats (280±20 g) were randomly divided into three groups, and were deeply anesthetized intraperitoneally with chloral hydrate (30 mg/kg) and placed in a stereotaxic instrument (SR-6N, Tokyo, Japan). After scalp incision and exposure of the occipital bone, holes were drilled at coordinates of 1.0 mm posterior, 1.5 mm lateral to bregma and 4.2 mm in depth. Via each hole, a microinjector (10 *μ*l) was slowly implanted into the lateral ventricle of the brain, then a volume of 10 *μ*l solution containing either physiological saline or bilirubin (25, 50 *μ*mol/l) saline solution was administered slowly. At 24 h after the intraventricular injection, the hippocampal tissue was collected for further studies.

### Morris water maze test

The spatial memory was blindly evaluated by a Morris water maze test. Before each experiment, the rats were brought to the site to allow them to be acclimatized. The temperature of the room and the water was kept at 24±2 °C. For spatial learning, the rats were trained to find a hidden platform for 6 consecutive days, 4 trials per day with a 20- to 30-s interval for each rat from 1500 to 2000 hours. On each trial, the rat started from one of the four middle quadrants facing the wall of the pool and ended when the animal climbed on the platform. The rats were not allowed to search for the platform for more than 60 s. They were guided to the platform if they could not find the platform within 60 s. Through these training sessions, rats acquired spatial memory about the location of the safe platform. The swimming pathways and the latencies of the rats to find the hidden platform were recorded each day. The pathway and the length that the rats passed through the previous platform quadrant were recorded by a video camera fixed to the ceiling of the room, 1.5 m from the water surface. The camera was connected to a digital-tracking device attached to an IBM computer loaded with the water maze software (Huaibei Zhenghua Biologic Apparatus Facilities, Anhui, China). The spatial memory was tested 48 h later after the last training. When the platform was withdrawn, the path was recorded; the longer a rat stayed in the quadrant where previously the platform had been located, the better it scored for the spatial memory (the percentage of distance traveled in the target quadrant and the escape latency were used to indicate the score).

### Primary cell culture

Hippocampal neurons were prepared from 1-day-old neonatal rats for primary cell cultures. In brief, the rats’ hippocampus were isolated in calcium-free Hanks’ balanced salt solution, incubated with 0.25% trypsin at 37 °C for 15 min, and triturated with a Pasteur pipette, the cell suspension was filtered through a nylon mesh (200 meshes), then centrifuged at 210 × *g* for 5 min. The cells were suspended in Neurobasal medium supplemented with B27, GlutaMAX, 100 U/ml penicillin, 100 *μ*g/ml streptomycin, and plated into PDL (0.1 mg/ml) coated 12-well plates. Cells were grown in humidified atmosphere of 95% air and 5% CO_2_. Medium was exchanged after 24 h and renewed every 3 days during the culturing period.

### Sample collection from human subjects and the isolation of human blood cells

Serum or peripheral blood samples of normal control individuals were obtained from Guangzhou Blood Center and the serum or peripheral blood samples of patients with digestive disease or newborns were obtained from discarded material utilized for routine laboratory tests at the Departments of Digestive Disease or Neonatology, Guangzhou First Municipal People’s Hospital of Guangzhou Medical University. The use of these materials is approved by the ethics committee of these two institutions and is with the permission of the patients and volunteers. The clinical diagnosis of physiological *versus* pathological jaundice in neonates was based on commonly accepted criteria for Chinese. In brief, physiological jaundice occurs in healthy newborns and it does not appear until 48 h after birth with the serum UCB level being below 12 mg/dl, whereas neonatal pathological jaundice appears within first 24 h postnatal or with a serum bilirubin level exceeding 12 mg/dl or rising at a rate over 5 mg/dl/day. For physiological jaundice, the serum bilirubin level returns normal by the end of 2 week after birth in the full term birth or 3 week with preterm newborns, whereas in pathological jaundice, the bilirubin level persists beyond the age for disappearance in term or preterm infants.^35, 36^

### Cell viability assay

MTS assay (CellTiter 96 Aqueous One Solution reagent) was used to test cell viability as we previously reported.^37^ In brief, 24 h after two intraperitoneal injections of bilirubin (50 or 100 *μ*g/g), hippocampal tissues were extracted from the newborn rats to conduct primary neuron cell culture. Cells were seeded at 2500 cells per well in a 96-well plate. After incubation for the indicated times, 20 *μ*l MTS reagent was directly added to each well and the incubation was continued for additional 3 h. The absorbance of optical density was measure with a microplate reader (Sunrise, Tecan, Männedorf, Switzerland) at wavelength 490 nm. Cell viability was calculated using the following formula: cell viability (%)=(average absorbance of treated group–average absorbance of blank)/(average absorbance of untreated group–average absorbance of blank) × 100%.

### Western blot analysis

Whole-cell lysates and rat hippocampal tissue homogenates were prepared in RIPA buffer (1 × PBS, 1% NP-40, 0.5% sodium deoxycholate, 0.1% SDS) supplemented with 10 mM *β*-glycerophosphate, 1 mM sodium orthovanadate, 10 mM NaF, 1 mM phenylmethylsulfonyl fluoride (PMSF) and 1 × Roche Complete Mini Protease Inhibitor Cocktail (Roche, Indianapolis, IN, USA). SDS-PAGE, transferring and immunodetection were performed as previously described.^37^ In brief, equal amounts of total protein extracts were fractionated by 12% SDS-PAGE and electrically transferred onto a polyvinylidene difluoride (PVDF) membrane. Primary antibodies and appropriate horseradish peroxidase-conjugated secondary antibodies were used to detect the designated proteins. The bound secondary antibodies on the PVDF membrane were reacted to the ECL detection reagents (Santa Cruz Biotechnology Inc.) and detected by exposing to X-ray films (Kodak, Rochester, NY, USA)..

### Peptidase activity assay

Fluorogenic substrate Suc-LLVY-AMC, Boc-LRR-AMC and Z-Leu-Leu-Glu-AMC were used to assess CT-like, trypsin-like, caspase-like activity of the 20S proteasome, respectively. To evaluate *in vivo* proteasome inhibition, cells were treated with bilirubin for different times at 37 °C. The drug-treated cells were then incubated with the Glo Cell-Based Assay Reagent (Promega Bioscience) for 10 min. Luminescence generated from each reaction was detected with microplate reader (Varioskan Flash 3001, Thermo, USA). To assay for direct inhibition of the 20S proteasome *in vitro*, purified human 20S proteasomes were incubated with the agent to be tested for 60 min at 37 °C before the addition of the fluorogenic substrate. Fluorescence intensity was measured using a spectrophotometer at excitation of 350 nm and emission of 438 nm (Varioskan Flash 3001, Thermo, Waltham, MA, USA).

### Molecular docking study

In order to obtain valuable binding information of bilirubin toward the DUBs mainly including USP14 and UCHL5, molecular docking studies were performed with CDOCKER protocol of Discovery Studio 2.0. (Accelrys Software Inc., San Diego, CA, USA). The crystallographic structures of USP14 and UCHL5 were directly downloaded from the Protein Data Bank (PDB IDs: 2AYO and 3RIS). After removing irrelevant components, hydrogen atoms were added and their positions were minimized with a 0.01 kcal/mol/Å root mean square gradient by using the all-atom CHARMm force-field and the Adopted Basis Newton-Raphson (NR) Algorithm. In addition, taking into account the dissociation of carboxylic acid in certain physiological condition (pH=7.35), the bilirubin anion was selected as the docking ligand. During the whole docking process, the two proteins were rigid, whereas the ligand was flexible. The Input Site Spheres of 10Å radius were centered on each active pocket of USP14 and UCHL5, with (x, y, z)=(39.65, 80.95, −1.239) and (−8.760, 11.29, 70.22), respectively. The conformation corresponding to the lowest CDOCKER Interaction Energy was selected as the most probable binding conformation.

### DUB activity assay

This was performed as reported.^38, 39^ Briefly, cell lysate (5 *μ*g) or 26S proteasomes (25 nM) were dissolved in ice-cold DUB buffer (50 mM Tris-HCl (pH 7.5), 250 mM sucrose, 5 mM MgCl_2_ and 1 mM PMSF) and pretreated with bilirubin, biliverdin or NEM for 15 min, then incubated with Ub-AMC substrate in a 100 *μ*l reaction volume at 25 °C. AMC released from substrate cleavage was temporally recorded with a microplate reader (Varioskan Flash 3001, Thermo).

### DUB active site-directed labeling assays

Purified 26S proteasomes (25 nM) were dissolved in DUB buffer (25 mM Tris-HCl pH 7.4, 5 mM MgCl, 20 mM NaCl, 200 *μ*M ATP), then treated with agents for 10 min before they were incubated with HA-UbVS for 1 h at 37 °C, followed by boiling in the reducing sample buffer and fractionated via SDS-PAGE. After transferred to PVDF membranes, HA-UbVS labeled DUBs were immuno-detected using anti-HA antibodies.

### Ubiquitin chain disassembly

*In vitro* disassembly of purified polyubiquitin chains (K48-linked) was performed as described earlier.^38, 39^ Purified 26S proteasomes (25 nM) were pre-incubated with either vehicle or agents to be tested for 10 min *in vitro* before mixed with K48-linked polyubiquitin chains (1 mg) in the DUB reaction buffer for 30 min at 37 °C. The reaction product was then fractionated using SDS-PAGE, transferred to PVDF membrane and assessed for the extent of chain disassembly via immunodetection of ubiquitin.

### Immunofluorescence

Primary hippocampal neurons were seeded in 12-well plates containing PDL coverslips. Cells were treated and fixed with a 4% paraformaldehyde fixative solution at room temperature for 15 min and washed three times in PBS (pH 7.4) for 5 min each. Cells were then permeabilized with 2% Triton PBS (2 ml Triton/100 ml PBS) for 10 min and blocked with 5% BSA in PBS for 1 h at room temperature in a humid chamber, then incubated overnight at 4 °C with primary antibodies. Followed by incubation with appropriate fluorescent dyes conjugated secondary antibodies at room temperature for 1 h, cells were then incubated with 4, 6-diamidino-2-phenylindole (10.5 *μ*g/ml, Molecular Probes by Life Technologies, Thermo, Waltham, MA, USA) for 15 min. Finally, after 1% glycerin sealing piece, cells were observed using a fluorescence microscope (Leica, Wetzlar, Germany).

### Measurement of mitochondrial membrane integrity

The mitochondrial membrane potential (ΔΨm) of bilirubin-treated and -untreated cells was assayed using JC-1 staining as previously reported.^40^ Briefly, cells were incubated with bilirubin for 24 h and stained with JC-1 (5 pg/ml) for 30 min at 37 °C. Following the staining, the cells were washed with PBS twice, and then imaged with an inverted fluorescence microscope equipped with a digital camera (Axio Obsever Z1, Zeiss, Oberkochen, Germany).

### Histology and immunohistochemistry

At different experimental time point, rats were anesthetized and transcardially perfused with 200 ml normal saline solution rapidly, and fixed *in situ* by perfusion for 20 min at 4 °C with Zamboni’s solution containing 4% paraformaldehyde, 15% saturated picric acid and 24 mM NaH2PO4/ 126 mM Na2HPO4 (pH 7.2). The brain was removed from the skull of the fixed animals and post-fixed in the same Zamboni’s solution for another 12 h at 4 °C. Then coronal slices (20 um) were cut with a Vibratome (Leica, VT1000S).

Sections were mounted on glass slides and stained with the H-E protocol: the slides were immersed in hematoxylin solution for 2–5 min, washed in tap water for 1 min, separately concentrated hydrochloric acid alcohol, washed in tap water for 3–5 min, transferred to eosin for 20–30 s. Conventional dehydration, transparenting with xylene and mounting with neutral resins were performed. Morphological changes in the stained sections were examined with light microscopy.

The immunohistological procedures were as follows: sections were permeabilized with 0.3% H_2_O_2_ in absolute methanol for 10 min to block endogenous peroxidase, and nonspecific sites were blocked with 5% BSA for 30 min at room temperature and incubated with primary antibodies for 24 h at 4 °C. The primary antibodies were used as indicated. After washing with PBS, sections were subsequently incubated with biotin-labeled secondary antibodies for 1 h at 37 °C. The immunoreaction was detected using horseradish peroxidase-labeled streptavidin for 1 h at 37 °C and visualized with the DAB tetrachloride system for brown color (Olympus Optical, Tokyo, Japan). A negative control for every antibody was used with PBS.

### Statistical analysis

All the results were expressed as mean±S.D. where applicable. GraphPad Prism 4.0 software (GraphPad Software Inc., La Jolla, CA, USA) was used for statistical analysis. Differences between two groups were evaluated for statistical significance using two-tailed Student’s *t*-test. A difference among three or more groups, one-way ANOVA or when appropriate, two-way ANOVA, followed by the Holm–Sidak test for pair-wise comparisons were performed. *P*-value of <0.05 was considered statistically significant.
